# Implementation Science: Bridging the Gap between Point-of-Care Diagnostics Research and Practice

**DOI:** 10.3390/diagnostics12071648

**Published:** 2022-07-06

**Authors:** Tivani P. Mashamba-Thompson

**Affiliations:** Faculty of Health Sciences, University of Pretoria, Pretoria 0002, South Africa; tivani.mashamba-thompson@up.ac.za

The advent of the novel Coronavirus 2019 (COVID-19) pandemic has fuelled technological innovation and led to the increased research on development and deployment of new diagnostics for use at point-of-care (POC). The rapid uptake of the newly developed diagnostics requires a systematic approach to bridge the research-to-practice gap. Implementation science (IS) involves the use of evidence-based practices (EBPs) that are characterised by both quality improvement and dissemination methods aiming to promote the scaling up of health interventions such as POC diagnostics to enhance quality and outcomes [1]. This research approach employs transdisciplinary quantitative and qualitative designs with solid grounding in theory. Implementation science studies are designed to enable identification of factors that impact uptake of health interventions across multiple levels, including the patient, provider, clinic, facility, organisation, and often the broader community and policy environment. In this Special Issue, we present a summary of twelve studies that employed implementation science approaches demonstrating research aimed at optimising implementation various kinds of point-of-care (POC) diagnostics among different population groups and different healthcare settings globally.

Nucleic acid amplification tests (NAATs), such as the reverse-transcriptase polymerase chain reaction (RT-PCR) tests, were the first to be developed and widely deployed at the beginning of the COVID-19 pandemic. These tests were designed to detect viral RNA. A RT-PCR-positive result is highly specific for the presence of viral nucleic acid. A study on portable, easy-to-use SARS-CoV-2 RT-PCR POC diagnostic device showed that the POC test’s performance was comparable to that of the conventional RT-qPCR tests [2]. It also showed that the POC RT-PCT displayed the characteristics of a POC test, with a short turnaround time for quick patient isolation. The implementation of the first official approved self-administered rapid antigen tests (RATs), CoviSelf, was assessed as part of a community-level COVID-19 pandemic response in rural India [3]. Results of the study show that self-administered RATs have potential in rural settings as they are cheap, quick and reasonably reliable. However, the tests kits were found to not be user-friendly and required equitable distribution to minimise the spread of COVID-19. One study conducted at a hospital setting in Germany evaluated the lung ultrasound (LUS) in 101 symptomatic patients with suspected COVID-19 infection at hospital admission [4]. Results of this evaluation demonstrate that early LUS examination as part of in-patient admission provides a diagnostic gain and is valuable for the clarification of SARS-CoV-2-suspected patients at hospital admission. An assessment of the correlation between hospital-based lung ultrasound (LUS) and chest X-ray (CXR) findings in 247 COVID-19 patients in Spain showed positive results [5]. A significant correlation between LUS findings and CXR in patients with suspected or confirmed COVID-19 infection and supported use of the LUS exam as the initial POC diagnostic imaging test was demonstrated.

Interventions such as mobile Health (mHealth) have been shown to help enhance health service delivery and access to disease diagnosis. Increased availability and use of mHealth to help improve access to diagnostics is recommended for resource-limited settings. Osei et al., 2021 conducted a study to examine the availability and use of mHealth for disease diagnosis and treatment support by healthcare professionals in the Ashanti Region of Ghana [6]. The findings in this study show minimal use of mHealth for disease diagnosis and treatment support by healthcare professionals at rural clinics, which in turn affects the accessibility of medical care in such resource constrained settings. One of the contributing factors for equitable accessibility, distribution and availability of POC tests is the agility of supply chain management systems. Maluleke et al., 2021, conducted a scoping review to systematically map evidence on supply chain management systems for POC diagnostics services with a focus on optimising the SARS-CoV-2 testing capacity in resource-limited settings [7]. The results of the review showed has showed that there is limited research evidence on POC diagnostics supply chain management systems globally. In addition to ensuring accessibility of new POC diagnostics to those who need it, acceptance by stakeholders is key to enabling uptake and appropriate usage of available diagnostics. Thirty-one stakeholders involved in adoption of POC ultrasound (POCUS) implementation in a US academic medical centre were interviewed to determine their perspective on this diagnostic intervention [8]. The Practical Robust Implementation and Sustainability Model (PRISM) was employed to guide framework analysis for the data collected from interviews with stakeholders. Stakeholders considered the following overarching themes to be important for the adoption and fidelity of POCUS by clinicians and health systems: clinical impact; efficiency; cost; development of credentialing policies; and robust quality assurance processes.

The need to optimise and monitor POC diagnostics quality management systems has been emphasised in other studies [9,10,11]. A systematic review and meta-analysis of global evidence showed moderate accuracy of mobile-linked POC diagnostics in detecting infections and recommended the development and deployment of more highly accurate mHealth-linked POC diagnostics [11]. Evaluation of the prognostic capacity of ΔLA (delta lactate) (correlation between prehospital lactate (pLA) and hospital lactate (hLA)) with respect to in-hospital two-day mortality among emergency department patients was also performed. Results of the evaluation demonstrate that lactate clearance in the initial moments of ED care appears to be a more reliable prognostic index than a baseline lactate value taken alone [9]. Hahn et al., 2021, employed a theorical model to demonstrate sensitivity-optimised screening as a “diagnostics as prevention” strategy for managing infectious diseases using HIV infections as a prototype [10]. The model was designed to increase case definitions for diagnostic test sensitivity of by compensating for the limited sensitivity of a test in the early stage of a disease. The model also enabled inclusion of known symptoms of the respective disease stage and RDT-based exposition prevention in a pandemic. This concept was widely used for the management of COVID-19 through applying rapid diagnostic tests with imperfect diagnostic accuracy.

Despite the technological advancements presented by the COVID-19 pandemic, disruption to services directed at management of existing infectious and deadly pandemics, such as TB was reported during the early stages of the pandemic. Dlangalala et al., 2021 systematically mapped available evidence on TB services at the primary healthcare (PHC) level during the COVID-19 period using as scoping review study [12]. The study revealed that pandemic mitigation strategies, as well as the fear and stigma experienced at the beginning of the pandemic, may have limited uptake of TB diagnostic services at PHC level. The presented poor TB service up-take may also be a result of poor health literacy, which has been shown to be generally low among vulnerable populations. In this context, health literacy would be defined in line with access to technology enabling disease screening, diagnosis and linkage to care, i.e., diagnostics literacy. The COVID-19 pandemic has revealed diagnostics literacy as one of the unmet needs among vulnerable populations that continue to experience short- and longer-term socio-economic consequences. To address this unmet need a multi-level diagnostics literacy advocacy model was proposed to help improve diagnostic uptake among vulnerable populations [13]. Sustainable implementation of the proposed model will require involvement of all key stakeholders.
